# Effect of Desulfurization Process Variables on the Properties of Crumb Rubber Modified Asphalt

**DOI:** 10.3390/polym14071365

**Published:** 2022-03-28

**Authors:** Honggang Zhang, Yangpeng Zhang, Jie Chen, Wenchang Liu, Wensheng Wang

**Affiliations:** 1 School of Traffic and Transportation Engineering, Changsha University of Science and Technology, Changsha 410114, China; 2 Guangxi Transportation Science and Technology Group Co., Ltd., Nanning 530007, China; cj_engineering@163.com; 3 Guangxi Key Lab of Road Structure and Materials, Nanning 530007, China; 4 School of Materials Science and Engineering, Chang’an University, Xi’an 710064, China; lwc_engineering@163.com; 5 College of Transportation, Jilin University, Changchun 130025, China; wangws@jlu.edu.cn

**Keywords:** asphalt, waste crumb rubber, desulfurization process, mechanochemical method

## Abstract

A large number of waste tires are in urgent need of effective treatment, and breaking waste tires into crumb rubber powder for modifying asphalt has been proved as a good idea to solve waste tires. Crumb rubber modified asphalt not only has good high and low temperature performance, durability, and aging resistance but can also reduce pavement noise and diseases, which has wide application prospects. In this study, crumb rubber powder was desulfurized by mechanochemical method to prepare desulfurized crumb rubber modified asphalt. During the desulfurization process of crumb rubber, the effects of desulfurization process variables including desulfurizer type, desulfurizer content, and desulfurization mixing temperature and time were considered, and then the physical properties of modified asphalt were tested. The test results showed that after mixing crumb rubber powder with desulfurizer, the viscosity of crumb rubber powder modified asphalt can be reduced. Moreover, the storage stability of crumb rubber powder modified asphalt could also be improved by mixing crumb rubber with desulfurizer. Based on the physical properties of crumb rubber powder modified asphalt, the desulfurization process of selected organic disulfide (OD) desulfurizer was optimized as follows: the OD desulfurizer content was 3%, the desulfurization mixing temperature was 160 °C, and the mixing time was 30 min. In addition, Fourier infrared spectroscopy analysis was carried out to explore the modification mechanism of desulfurized crumb rubber powder modified asphalt. There is no fracture and formation of chemical bonds, and the modification of asphalt by crumb rubber powder is mainly physical modification.

## 1. Introduction

With the rapid development of the rubber industry and automobile industry, a large number of waste rubber tire products have piled up [1,2,3,4]. In China, the amount of waste tires has reached 13.07 million tons by 2016, and it is still growing rapidly every year. It is expected that the accumulation of waste tires in China will rank first in the world [5,6,7]. The accumulation of waste tires is not only a waste of rubber resources but also a serious harm to the environment [8,9,10]. Therefore, the recycling of waste rubber has important social significance and economic value.

It has been found that adding crumb rubber powder as a modifier into asphalt for modification can effectively improve the technical properties of bitumen, thus that the road performance and service quality of the modified asphalt mixture can meet the requirements of road traffic operations [11,12,13]. Crumb rubber modified asphalt mixture has excellent high and low temperature performance, weatherability, elasticity, and anti-aging properties, can reduce pavement thickness, reduce traffic noise, and effectively prevent freezing in cold areas, and prolong the service life of the pavement [14,15,16]. On the other hand, there is excellent field performance exhibited by the dry process rubber modification of asphalt mixtures, which is equivalent to polymer-modified mixtures [17]. Picado-Santos et al. studied the functional characteristics, including roughness, skid resistance, texture, and a quality index for a crumb rubber asphalt pavement produced by the dry process, that had been open to traffic for eight years after rehabilitation. The comparison results showed that crumb rubber asphalt pavement produced by the dry process behaved in a very robust way [18]. Rodriguez-Fernandez et al. analyzed the dry process for the incorporation of crumb rubber from waste tires and found that mixtures with crumb rubber have adequate performance, being less susceptible to aging than a conventional polymer-modified mixture [19]. Rath et al. investigated the effects of a chemically engineered dry-process ground tire rubber modification in bitumen and mixtures, and the cracking and rutting performance of all the field sections was good-to-excellent [20,21]. However, for wet-process modified rubber asphalt, because the crumb rubber powder used in ordinary crumb rubber modified asphalt was treated by a vulcanization process, it had a relatively solid three-dimensional network structure and low activity, which makes the crumb rubber powder difficult to be compatible in asphalt and often accompanied by a large number of crumb rubber particles [22,23,24]. The ordinary crumb rubber modified asphalt prepared from vulcanized crumb rubber powder has a series of shortcomings, such as poor high temperature storage stability, easy segregation, high viscosity, and difficult construction, which limits its development and application [25,26,27]. Relevant research shows that rubber powder with high surface activity can be obtained by using desulfurization technology, leading to the reduction of viscosity of vulcanized crumb rubber powder modified asphalt [23]. The desulfurized crumb rubber modified asphalt has been proved to have good storage stability, low-temperature performance, and workability. In addition, the harmful gas emission of desulfurized crumb rubber modified asphalt is lower, thus it is more environmentally friendly [24,28]. Therefore, it is necessary to desulfurize and activate the vulcanized crumb rubber powder to improve the stability of crumb rubber modified asphalt.

In view of the shortcomings of ordinary crumb rubber modified asphalt, researchers carried out desulfurization activation treatment on crumb rubber powder to a certain extent, and then prepared desulfurized crumb rubber modified asphalt [28,29]. The earliest desulfurized crumb rubber modified asphalt can be traced back to the 1970s, which was tested and prepared in America. Later, a large number of studies were involved in this aspect; Ye et al. observed the microstructure of ordinary crumb rubber powder, dynamic desulfurized crumb rubber powder, and high-speed shear desulfurized crumb rubber powder in asphalt through fluorescence microscope, and detected the properties of three modified asphalt [30]. The results showed that dynamic desulfurization could destroy the internal vulcanization structure of crumb rubber and improve the compatibility between crumb rubber powder and asphalt, but its performance is lower than that of ordinary crumb rubber modified asphalt. High-speed shear desulfurization can not only improve the swelling capacity of asphalt crumb rubber powder but also avoid agglomeration. Therefore, the performance of asphalt can be improved by high-speed shear desulfurized crumb rubber powder. Ibrahim et al. used the waste rubber treated by gamma rays to produce crumb rubber asphalt [31]. Through comparison, it was found that the properties of crumb rubber modified asphalt made by gamma radiation treated waste rubber was increased significantly at a higher temperature, lower temperature, and aging resistance than that of untreated crumb rubber. Lin et al. studied the effect of the dissolution of crumb rubber on the physical properties of asphalt under the reaction conditions of high temperature and high shear rate [32]. The results showed that under the condition of high temperature reaction, crumb rubber molecules dissolved rapidly, degraded, and depolymerized, thus as to reduce its average molecular weight. It is speculated that ordinary crumb rubber powder may produce desulfurization reaction under high temperature, but when the temperature is too high, it will lead to the aging of crumb rubber modified asphalt. Ma et al. studied the modification mechanism of desulfurized crumb rubber modified asphalt by using scanning electron microscope, component analysis, and infrared spectroscopy, and tested the road performance of its modified asphalt and mixture [33]. The results showed that the modification mechanism of desulfurized crumb rubber modified asphalt was different from that of ordinary crumb rubber modified asphalt, and the desulfurized crumb rubber powder can have an obvious chemical reaction with asphalt, which makes the performance of the two have significant differences. Compared with ordinary crumb rubber modified asphalt, desulfurized crumb rubber modified asphalt has a lower viscosity and better storage stability and is suitable for dense mixtures. Juganaru et al. modified asphalt with partially desulfurized crumb rubber [34]. Through fluorescence microscope observation, the distribution of desulfurized crumb rubber in asphalt was more uniform. Compared with vulcanized crumb rubber modified asphalt, the adhesion of desulfurized crumb rubber powder modified asphalt was significantly increased, which can improve the workability of rubber asphalt.

Many scholars have studied the desulfurization methods and desulfurization conditions of waste tire rubber powder, covering the desulfurization process and common desulfurization methods. The research indicated that high temperature, high-speed shear, gamma radiation, and other conditions could make the desulfurization of ordinary crumb rubber powder. After desulfurization, the spatial structure of crumb rubber powder was changed, and the molecular weight was reduced, which can increase the reaction degree with asphalt, significantly improve the compatibility between crumb rubber powder and asphalt, and effectively improve the defects of crumb rubber modified asphalt, such as easy segregation and poor high temperature storage stability. However, there are few studies on the production process of desulfurized crumb rubber powder modified asphalt and the road performance of modified asphalt mixture. At present, the commonly used rubber desulfurization activation methods include heat treatment based on the thermal environment, a chemical method using chemical adjuvants, and a mechanochemical method using chemical adjuvants combined with mechanical force to accelerate the chemical reaction [27,35,36]. The mechanochemical method can effectively play the role of desulfurization activation of additives through rubber broken to increase the surface roughness and activity of rubber powder. Moreover, the mechanochemical method has the advantages of simple equipment, high production efficiency, low cost, and small smell, and is very suitable for large factory production. Therefore, in this study, the crumb rubber powder was desulfurized by the mechanochemical method, and the desulfurized crumb rubber powder modified asphalt was prepared. The effects of the desulfurization process on the physical properties and micro characterization of crumb rubber powder modified asphalt were systematically studied.

## 2. Materials and Methods

### 2.1. Raw Materials

**Figure 1:**
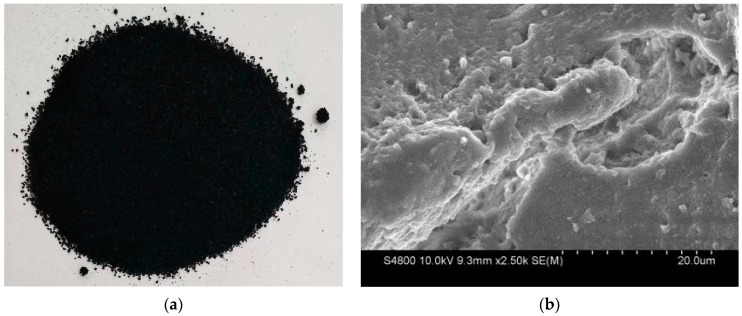
Crumb rubber used in this study: (**a**) original appearance; (**b**) SEM.

In this study, Maoming 70# road petroleum asphalt (Guangdong, China) was adopted, with a penetration of 72 (0.1 mm), softening point of 46.0 °C, ductility at 15 °C more than 100 cm, and density of 1.08 g/cm^3^. The crumb rubber powder was 30 mesh made of the waste tire of wheels, in which rubber hydrocarbon content was 48.9%, total organic content was 54.5%, sulfur content was 1.896%, and its density was 1.33 g/cm^3^. The original appearance and scanning electron microscope (SEM) of crumb rubber used in this study are shown in Figure 1 to better describe the macro and micromorphology of crumb rubber. The desulfurizer used in this study (Hebei Richway Technology Co. Ltd., Shijiazhuang, China) includes adjuvant (organometallic complexes) labeled as OMC, adjuvant (organic disulfide) labeled as OD, and self-made desulfurizer (deep eutectic solvents) labeled as DES, which have the advantages of better desulfurization effect, stable desulfurizer source, and simple desulfurization process [37,38].

### 2.2. Sample Preparation

The modified crumb rubber powder by mechanochemical method was prepared using an internal mixer according to previous studies [37,38]. After adding crumb rubber powder into the internal mixing chamber, appropriately increasing the temperature and maintaining low-speed rotation, the desulfurizer was also added into the internal mixing chamber until it was mixed evenly. Then, the mixing temperature and mixing time was set, and the desulfurized crumb rubber powder could be prepared at a higher rotation speed after the internal mixer stopped.

In order to prepare desulfurized crumb rubber modified asphalt, the following steps were adopted:

1. Place the base asphalt in an oven of 140 °C and heat it to the flowing state, and slowly add the weighed desulfurized crumb rubber powder (25% of asphalt by mass) with a mixing speed of 300 r/min. During the addition process, the temperature was raised to 170 °C and maintained for 10 min;
2. Put the mixed asphalt an oven of 170 °C and swell for 40 min;
3. The swelled rubber asphalt was placed to a high-speed shear equipment, the shear rate was gradually increased to about 4500 r/min, and the temperature was controlled at 185 °C, and then taken it out after shearing for 1 h;
4. Put the prepared crumb rubber modified asphalt into an oven of 175 °C for 30 min, and then the crumb rubber modified asphalt samples could be used for test according to the specification requirements.

### 2.3. Experimental Methods

In this study, according to the Chinese standard “Standard Test Methods of Bitumen and Bituminous Mixtures for Highway Engineering” (JTG E20-2011), the penetration, softening point, ductility at 5 °C, and rotational viscosity at 180 °C was tested for crumb rubber powder modified asphalt [39]. In addition, the Fourier infrared spectrum of these materials was also tested [40].

## 3. Results and Discussion

### 3.1. Comparison and Selection of Desulfurizer Type Based on Physical Properties of Crumb Rubber Modified Asphalt

Referring to the recommended process in the desulfurizer product manual, the mixing time of crumb rubber modified asphalt was 60 min, the mixing temperature was 120 °C, and the desulfurizer content was 3%. The penetration, ductility, softening point, rotational viscosity at 180 °C, elastic recovery, and softening point difference (Δ) of the crumb rubber modified asphalt were tested according to the “Standard Test Methods of Bitumen and Bituminous Mixtures for Highway Engineering” (JTG E20-2011), and the effect of desulfurizer types on the physical properties of crumb rubber modified asphalt could be studied to compare and select a better adjuvant for in-depth and detailed process variable analysis. The results are shown in Table 1.

**Figure 2:**
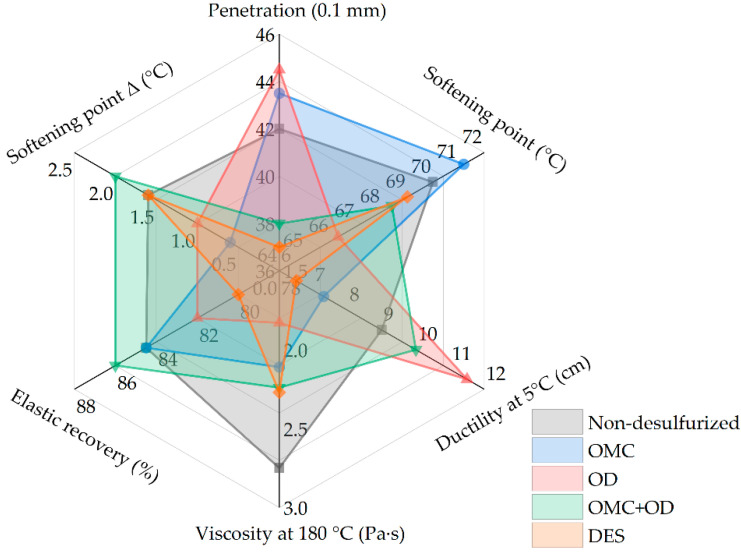
Radar chart for of desulfurizer type comparison based on physical properties of crumb rubber modified asphalt.

It can be seen from Table 1 that the viscosity results at 180 °C of modified asphalt prepared by desulfurized crumb rubber powder treated with OMC, OD, and DES were reduced. The penetration values of modified asphalt with desulfurized crumb rubber powder treated with OMC and OD were higher than that of ordinary non-desulfurized crumb rubber modified asphalt, while the desulfurizer (OMC + OD or DES) could reduce the penetration of modified asphalt. Except that the softening point value of modified asphalt with desulfurized crumb rubber powder treated with OMC increased, the other softening point results of modified asphalt were all reduced. Besides, crumb rubber powder treated with desulfurizers OMC and DES could reduce the ductility at 5 °C of modified asphalt. The segregation softening point difference of desulfurized crumb rubber powder modified asphalt using OMC and OD alone were decreased after 48 h of storage. Thus, the purpose of improving the compatibility between crumb rubber powder and asphalt and reducing the viscosity of crumb rubber modified asphalt through crumb rubber powder desulfurization technology can be achieved. According to the comparison results in the radar chart (Figure 2), OMC increased the penetration and softening point of desulfurized crumb rubber modified asphalt, while the ductility, viscosity at 180 °C, and softening point Δ were greatly reduced. For desulfurized crumb rubber modified asphalt by OD, except for the penetration and ductility, other physical properties decreased. However, for desulfurized crumb rubber modified asphalt by OMC + OD, the improvement effect of modified asphalt properties was not a simple superposition of modified asphalt using OMC and OD alone. DES reduced the overall physical properties of crumb rubber modified asphalt to varying degrees. Considering the two indexes of ductility at 5 °C and viscosity at 180 °C, the desulfurizer OD was selected as the desulfurizer used for the follow-up study.

### 3.2. Effect of Desulfurizer Content on the Properties of Crumb Rubber Modified Asphalt

**Figure 3:**
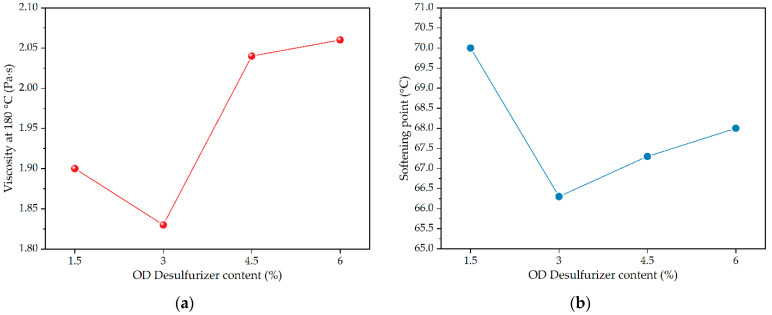

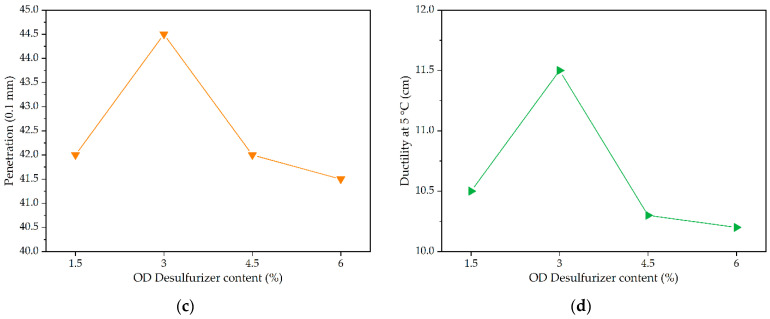
The physical properties of asphalt modified by crumb rubber treated with different OD desulfurizer contents: (**a**) viscosity at 180 °C; (**b**) softening point; (**c**) penetration; (**d**) ductility at 5 °C.

The desulfurized crumb rubber powder modified asphalt prepared by crumb rubber treated with different OD desulfurizer content (i.e., 1.5%, 3%, 4.5%, 6%) were prepared with the mixing time of 60 min and mixing temperature of 120 °C. The changes of physical properties of desulfurized crumb rubber powder modified asphalt were compared and analyzed. The comparison results are shown in Figure 3.

From Figure 3a,b, it can be seen that with the increase of OD desulfurizer content, the viscosity at 180 °C, and softening point of desulfurized crumb rubber powder modified asphalt had similar change laws; these two physical properties first decreased and then increased. When the OD desulfurizer content was 3%, the viscosity at 180 °C and softening point of desulfurized crumb rubber powder modified asphalt reached the minimum. On the other hand, with the increase of OD desulfurizer content, the penetration and ductility at 5 °C of desulfurized crumb rubber powder modified asphalt had similar change laws. In Figure 3c,d, the values of penetration and ductility increased first and then decreased. It was also found that the penetration and ductility at 5 °C of desulfurized crumb rubber powder modified asphalt reached the maximum at the OD desulfurizer content of 3%.

The reason for the above phenomena is that the OD desulfurizer can promote the desulfurization and activation of crumb rubber powder by opening the S-S and C-S keys in the cross-linked network, and partially restore the properties of raw rubber to improve the compatibility with asphalt [41]. The greater the amount of OD desulfurizer content, the more obvious the desulfurization effect of crumb rubber [42]. When the content of OD desulfurizer is too high, some OD desulfurizer will not participate in the desulfurization reaction with crumb rubber and have a surplus of OD desulfurizer. This surplus part of OD desulfurizer would interact with asphalt during the modification process of asphalt, resulting in a poor modification effect of crumb rubber modified asphalt, i.e., the too low penetration and ductility as well as too high viscosity as shown in Figure 3. Therefore, the content of OD desulfurizer needs to be controlled within a reasonable range. According to the above analysis results of the physical properties of crumb rubber modified asphalt, the recommended content of OD desulfurizer was set as 3%.

### 3.3. Effect of Mixing Temperature on the Properties of Crumb Rubber Modified Asphalt

**Figure 4:**
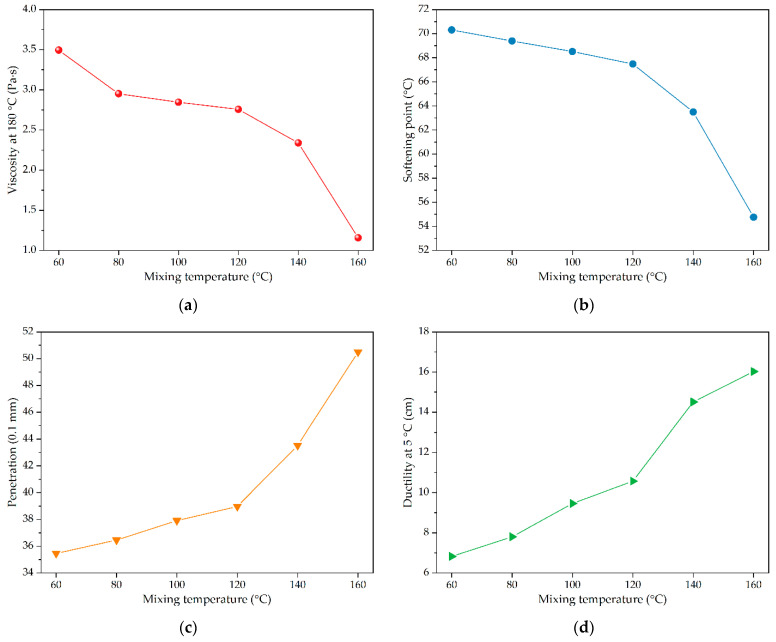
The physical properties of asphalt modified by crumb rubber treated with different mixing temperatures (i.e., 60, 80, 100, 120, 140, 160 °C): (**a**) viscosity at 180 °C; (**b**) softening point; (**c**) penetration; (**d**) ductility at 5 °C.

Based on the selected desulfurizer type of OD desulfurizer and its content of 3% of crumb rubber powder by mass, the effects of mixing process on the performances of crumb rubber modified asphalt were also studied. In order to ensure the reaction between crumb rubber powder and the OD desulfurizer at a lower temperature, the mixing time of crumb rubber powder was fixed for 90 min, the asphalt modified by crumb rubber powder treated with OD desulfurizer were prepared at different desulfurization temperatures (i.e., 60 °C, 80 °C, 100 °C, 120 °C, 140 °C, and 160 °C). The changes of properties of desulfurized crumb rubber powder modified asphalt were compared and analyzed, and the comparison results are shown in Figure 4.

In Figure 4a,b, it can be seen that with the increase of mixing temperature, the viscosity at 180 °C and softening point of desulfurized crumb rubber powder modified asphalt have similar change trends, and the viscosity at 180 °C and softening point of modified asphalt gradually decrease. For the mixing temperature between 60 °C and 120 °C, the decline of viscosity at 180 °C and softening point were moderate. While the mixing temperature reached 120 °C, the viscosity at 180 °C and softening point of desulfurized rubber powder modified asphalt were significantly reduced. This is because under the action of OD desulfurizer and mechanical force, the crumb rubber powder was desulfurized and activated, resulting in some properties of raw crumb rubber being restored to improve the compatibility with asphalt, and the particle core of crumb rubber powder became smaller [43]. Therefore, the high temperature performance of desulfurized crumb rubber powder modified asphalt became worse, and the corresponding viscosity decreased to a certain extent.

As shown in Figure 4c,d, there were also some similar variation trends of the penetration and ductility at 5 °C for desulfurized crumb rubber powder modified asphalt. When the mixing temperature was lower than 120 °C, the penetration of desulfurized crumb rubber powder modified asphalt gradually increased with the increase of mixing time. While the mixing temperature was higher than 120 °C, the penetration of desulfurized crumb rubber powder modified asphalt sharply increased. With the increase of mixing time, the ductility at 5 °C of desulfurized crumb rubber powder modified asphalt increased gradually. Through analysis, it can be known that under the action of OD desulfurizer and mechanical force, crumb rubber powder was desulfurized and activated with restored properties of raw crumb rubber to improve the compatibility with asphalt. After the desulfurized crumb rubber powder was used for modifying base asphalt, the consistency of desulfurized crumb rubber powder modified asphalt would be reduced and the corresponding ductility could be enhanced [44].

To sum up, when the mixing temperature was low, the desulfurization effect of crumb rubber powder was not obvious, and the viscosity reduction of desulfurized crumb rubber powder modified asphalt was too small. At the same time, the mixing temperature needs to be higher than 120 °C. Therefore, the mixing temperatures of 140 °C and 160 °C were selected as the mixing temperature for subsequent tests. Subsequently, desulfurized crumb rubber powder was prepared and modified base asphalt under the test conditions of mixing temperature of 140 °C and mixing time of 120 min. It was found that the viscosity of the modified asphalt decreased significantly, but the softening point also decreased significantly. At the same time, considering that the mixing time was too long, it was not suitable for large-scale production [45]. Therefore, the mixing temperature of 160 °C was selected as the preferred mixing temperature.

### 3.4. Effect of Mixing Time on the Properties of Crumb Rubber Modified Asphalt

**Figure 5:**
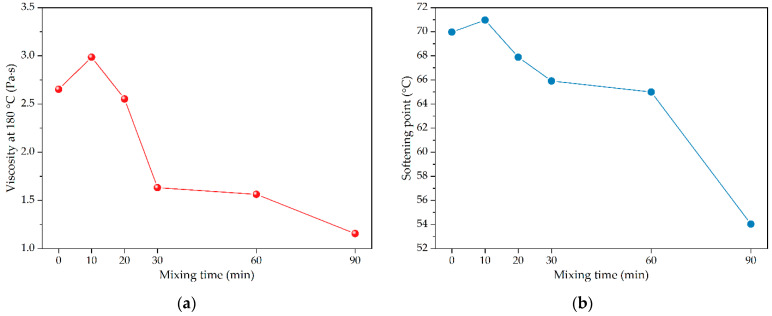

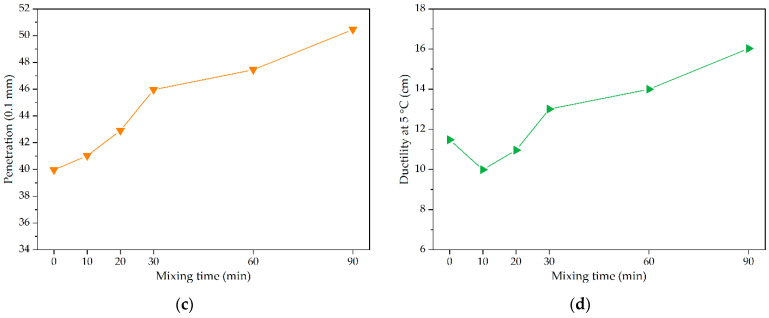
The physical properties of asphalt modified by crumb rubber treated with different mixing time (i.e., 0, 10, 20, 30, 60, 90 min): (**a**) viscosity at 180 °C; (**b**) softening point; (**c**) penetration; (**d**) ductility at 5 °C.

According to the above analysis results, the desulfurizer type was selected as OD desulfurizer, and its content was determined as 3% of crumb rubber powder by mass, the effects of mixing process on the performances of crumb rubber modified asphalt were further studied. The mixing temperature for desulfurized crumb rubber powder was preferably 160 °C, and the desulfurized crumb rubber powder modified asphalt under different desulfurization mixing times (i.e., 10 min, 20 min, 30 min, 60 min, and 90 min) were prepared. The changes of properties of desulfurized crumb rubber powder modified asphalt were compared and analyzed, and the corresponding comparison results are shown in Figure 5.

It can be seen from Figure 5a,b that with the increase of mixing time, the viscosity at 180 °C and softening point of desulfurized crumb rubber powder modified asphalt first increased and then decreased. However, when the mixing time was 30 min, the viscosity at 180 °C of desulfurized crumb rubber powder modified asphalt decreased greatly, while the softening point decreased only slightly. Therefore, the desulfurization mixing time of crumb rubber powder was determined as 30 min. The corresponding analysis shows that the existence of mechanical force can promote the chemical reaction between crumb rubber powder and the selected OD desulfurizer, and the crumb rubber powder was activated by OD desulfurization, partially restoring the properties of raw crumb rubber to improve the compatibility with asphalt [45]. When the mixing time was too short, the reaction between the OD desulfurizer and crumb rubber powder was insufficient, and some OD desulfurizer remained in desulfurized crumb rubber powder, which will react with asphalt, resulting in a poor modification effect of asphalt. However, when the mixing time is too long, the crumb rubber powder will be excessively reduced under the action of mechanical force, and the core of crumb rubber powder particles will lose strength, which will also lead to the deterioration of high temperature performance of modified asphalt [46].

As illustrated in Figure 5c,d, the penetration of desulfurized crumb rubber powder modified asphalt gradually increased with the increase of mixing time. The ductility at 5 °C of desulfurized crumb rubber powder modified asphalt first decreased and then increased. Through analysis, under the action of the OD desulfurizer and mechanical force, crumb rubber powder was desulfurized and activated, and some properties of raw rubber were restored to improve the compatibility with asphalt. After being used for the modification of asphalt, the consistency of desulfurized crumb rubber powder modified asphalt decreased and the ductility was enhanced. With the increase of mixing time, the degree of desulfurized activation of crumb rubber powder increased. After the modification of asphalt, the changes of the properties of desulfurized crumb rubber powder modified asphalt became more and more obvious with mixing time [47].

Combined with the analysis results of deterioration mixing temperature and mixing time, when the mixing temperature was 160 °C and the mixing time was 30 min, the overall properties of desulfurized crumb rubber powder modified asphalt could be regarded as the best, its viscosity decreased significantly, and the reduction of softening point was limited. Therefore, the preferred mixing process is a mixing temperature of 160 °C and mixing time of 30 min.

### 3.5. Micro Characterization of Desulfurized Crumb Rubber Modified Asphalt

**Figure 6:**
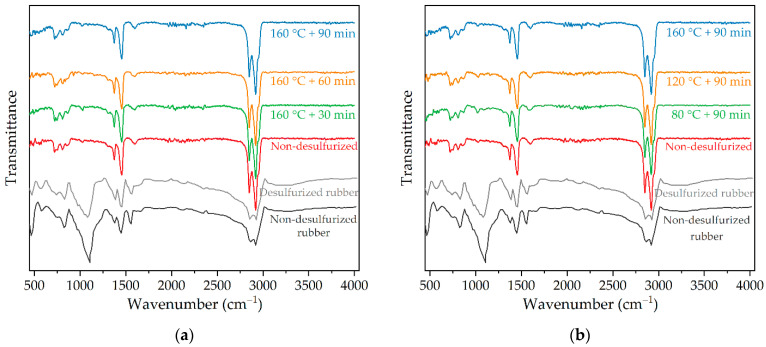
Fourier infrared spectroscopy of desulfurized crumb rubber powder modified asphalt: (**a**) with different mixing time; (**b**) with different mixing temperature.

In order to explore the modification mechanism of desulfurized crumb rubber powder modified asphalt, Fourier infrared spectroscopy analysis was carried out on desulfurized crumb rubber powder modified asphalt under different mixing temperatures and mixing times. The Fourier infrared spectroscopy results are shown in Figure 6.

From Figure 6, it can be seen that the Fourier infrared spectrum of crumb rubber powder modified asphalt mainly shows three concentrated strong absorption peaks [29], of which the absorption peaks at 1925 cm^−1^ and 2843 cm^−1^ were mainly caused by the stretching vibration of “-CH_2_-“ in cycloalkanes and alkanes of asphalt. The absorption peak at 1350~1470 cm^−1^ was caused by the bending vibration in the “C-H” bond plane, which was the shear vibration absorption peak of the “-CH_3_” alkane group in asphalt. The absorption peak at 1611 cm^−1^ was caused by the “C=C” bond and “C=O” bond, which is a typical characteristic peak of aromatic components in asphalt. The C-S bond was destroyed in the desulfurization process, and the “S=O” bond at 1082 cm^−1^ and the “C=O” bond at 1710 cm^−1^ were changed in the spectrum in varying degrees shown in Figure 6. However, the “S=O” bond and “C=O” bond do not exist in the spectrum of modified asphalt, which shows that some functional groups in the rubber reacted with asphalt, making their combination more stable. In addition, compared with different mixing time and mixing temperature, the position and peak of the characteristic peaks of the Fourier infrared spectrum of the desulfurized crumb rubber powder modified asphalt did not change significantly, which indicates that the fracture and formation of chemical bonds were not found in the modification process of desulfurized crumb rubber powder modified asphalt, and the modification process of asphalt by desulfurized crumb rubber powder was mainly physical blending, which is also consist with previous studies [4,48,49].

## 4. Conclusions

In this study, crumb rubber powder was desulfurized by mechanochemical method to prepare desulfurized crumb rubber modified asphalt. During the desulfurization process of crumb rubber, the effects of the desulfurization process variables including desulfurizer type, desulfurizer content, and desulfurization mixing temperature and time were considered, and then the physical properties of modified asphalt were tested.

(1) Two commercial regenerant labeled as OMC, activators labeled as OD, as well as self-made desulfurizer labeled as DES and crumb rubber powder were selected for mixing the desulfurization treatment. The three desulfurizers can reduce the viscosity of desulfurized crumb rubber powder modified asphalt, among which OD and OMC can reduce the softening point at the same time;

(2) The desulfurization process of selected OD desulfurizer is optimized as follows: the OD desulfurizer content is 3%, the desulfurization mixing temperature is 160 °C and the mixing time is 30 min;

(3) Based on the Fourier infrared spectrum analysis, the desulfurization treatment did not change the functional groups of crumb rubber powder modified asphalt, and the modification of asphalt by crumb rubber powder was mainly physical modification.

Future research on the performing and defining grade of each modified asphalt binder as well as the effects of desulfurization agents on rubber is highly warranted.

## Figures and Tables

**Table 1:** The physical properties of asphalt modified by crumb rubber treated with different desulfurizer types.

Types	Penetration (0.1 mm)	Softening Point (°C)	Ductility at 5 °C (cm)	Viscosity at 180 °C (Pa·s)	Elastic Recovery (%)	Softening Point Δ (°C)
Non-desulfurized crumb rubber modified asphalt	42.0	70.0	9.0	2.75	84.5	1.6
Crumb rubber modified asphalt (Desulfurized by OMC)	43.5	71.2	7.3	2.11	84.5	0.6
Crumb rubber modified asphalt (Desulfurized by OD)	44.5	66.3	11.5	1.83	82.0	1.0
Crumb rubber modified asphalt (Desulfurized by OMC + OD)	38.0	68.4	10.0	2.24	86.0	2.0
Crumb rubber modified asphalt (Desulfurized by DES)	37.0	69.0	6.5	2.27	80.0	1.6

## Data Availability

Not applicable.

## References

[1] Wang W.S., Cheng Y.C., Chen H.P., Tan G.J., Lv Z.H., Bai Y.S. (2019). Study on the performances of waste crumb rubber modified asphalt mixture with eco-friendly diatomite and basalt fiber. Sustainability.

[2] Saberi K.F., Fakhri M., Azami A. (2017). Evaluation of warm mix asphalt mixtures containing reclaimed asphalt pavement and crumb rubber. J. Clean. Prod..

[3] Ge D.D., Zhou X.D., Chen S.Y., Jin D.Z., You Z.P. (2020). Laboratory evaluation of the residue of rubber-modified emulsified asphalt. Sustainability.

[4] Chen T., Ma T., Huang X.M., Guan Y.S., Zhang Z.X., Tang F.L. (2019). The performance of hot-recycling asphalt binder containing crumb rubber modified asphalt based on physiochemical and rheological measurements. Constr. Build. Mater..

[5] Chen M.Z., Zheng J., Li F.Z., Wu S.P., Lin J.T., Wan L. (2015). Thermal performances of asphalt mixtures using recycled tyre rubber as mineral filler. Road Mater. Pavement Des..

[6] Ding X.H., Ma T., Zhang W.G., Zhang D.Y. (2017). Experimental study of stable crumb rubber asphalt and asphalt mixture. Constr. Build. Mater..

[7] Yu H.Y., Leng Z., Zhou Z.Y., Shih K.M., Xiao F.P., Gao Z.M. (2017). Optimization of preparation procedure of liquid warm mix additive modified asphalt rubber. J. Clean. Prod..

[8] Marini S., Lanotte M. (2021). Waste rubber from end-of-life tires in ‘lean’ asphalt mixtures-a laboratory and field investigation in the arid climate region. Polymers.

[9] Chen S.Y., Ge D.D., Jin D.Z., Zhou X.D., Liu C.C., Lv S.T., You Z.P. (2020). Investigation of hot mixture asphalt with high ground tire rubber content. J. Clean. Prod..

[10] de Almeida A.F., Battistelle R.A., Bezerra B.S., de Castro R. (2012). Use of scrap tire rubber in place of sbs in modified asphalt as an environmentally correct alternative for brazil. J. Clean. Prod..

[11] Kukielka J., Bankowski W., Mirski K. (2021). Asphalt-cement concretes with reclaimed asphalt pavement and rubber powder from recycled tire. Materials.

[12] Wang S.Q., Gao Y., Yan K.Z., You L.Y., Jia Y.S., Dai X.W., Chen M., Diab A. (2021). Effect of long-term aging on waste tire rubber and amorphous poly alpha olefin compound modified asphalt binder and its mixtures. Constr. Build. Mater..

[13] Li D.N., Leng Z., Zou F.L., Yu H.Y. (2021). Effects of rubber absorption on the aging resistance of hot and warm asphalt rubber binders prepared with waste tire rubber. J. Clean. Prod..

[14] Wang H.N., You Z.P., Mills-Beale J., Hao P.W. (2012). Laboratory evaluation on high temperature viscosity and low temperature stiffness of asphalt binder with high percent scrap tire rubber. Constr. Build. Mater..

[15] Kocak S., Kutay M.E. (2020). Fatigue performance assessment of recycled tire rubber modified asphalt mixtures using viscoelastic continuum damage analysis and aashtoware pavement me design. Constr. Build. Mater..

[16] Yan C., Lv Q., Zhang A.A., Ai C., Huang W., Ren D. (2022). Modeling the modulus of bitumen/sbs composite at different temperatures based on kinetic models. Compos. Sci. Technol..

[17] Buttlar (2021). State of Knowledge Report on Rubber Modified Asphalt.

[18] Picado-Santos L.G., Capitao S.D., Dias J.L.F. (2019). Crumb rubber asphalt mixtures by dry process: Assessment after eight years of use on a low/medium trafficked pavement. Constr. Build. Mater..

[19] Rodriguez-Fernandez I., Cavalli M.C., Poulikakos L., Bueno M. (2020). Recyclability of asphalt mixtures with crumb rubber incorporated by dry process: A laboratory investigation. Materials.

[20] Rath P., Majidifard H., Jahangiri B., Chen S., Buttlar W.G. (2021). Laboratory and field evaluation of pre-treated dry-process rubber-modified asphalt binders and dense-graded mixtures. Transp. Res. Rec..

[21] Rath P., Meister J., Jahangiri B., Buttlar W. (2022). Evaluation of the effects of engineered crumb rubber (ecr) on asphalt mixture characteristic. J. Test. Eval..

[22] Ghabchi R., Arshadi A., Zaman M., March F. (2021). Technical challenges of utilizing ground tire rubber in asphalt pavements in the united states. Materials.

[23] Liu W.H., Xu Y.S., Wang H.J., Shu B.N., Barbieri D.M., Norambuena-Contreras J. (2021). Enhanced storage stability and rheological properties of asphalt modified by activated waste rubber powder. Materials.

[24] Xu P., Gao J.P., Pei J.Z., Chen Z., Zhang J.P., Li R. (2021). Research on highly dissolved rubber asphalt prepared using a composite waste engine oil addition and microwave desulfurization method. Constr. Build. Mater..

[25] Liu B.Q., Li J., Han M.Z., Zhang Z.Q., Jiang X.Y. (2020). Properties of polystyrene grafted activated waste rubber powder (ps-arp) composite sbs modified asphalt. Constr. Build. Mater..

[26] Chen Z.X., Pei J.Z., Wang T., Amirkhanian S. (2019). High temperature rheological characteristics of activated crumb rubber modified asphalts. Constr. Build. Mater..

[27] Yang X.L., Shen A.Q., Li B., Wu H.S., Lyu Z.H., Wang H., Lyu Z.F. (2020). Effect of microwave-activated crumb rubber on reaction mechanism, rheological properties, thermal stability, and released volatiles of asphalt binder. J. Clean. Prod..

[28] Wang J.R., Zhang Z.Q., Li Z.L. (2020). Performance evaluation of desulfurized rubber asphalt based on rheological and environmental effects. J. Mater. Civil. Eng..

[29] Ma T., Zhao Y.L., Huang X.M., Zhang Y. (2016). Characteristics of desulfurized rubber asphalt and mixture. Ksce J. Civ. Eng..

[30] Ye Z.G., Kong X.M., Yu J.Y., Wei L.Q. (2003). Microstructure and properties of desulfurized crumb rubber modified bitumen. J. Wuhan Univ. Technol..

[31] Ibrahim I.M., Fathy E.S., El-Shafie M., Elnaggar M.Y. (2015). Impact of incorporated gamma irradiated crumb rubber on the short-term aging resistance and rheological properties of asphalt binder. Constr. Build. Mater..

[32] Lin P., Huang W.D., Tang N.P., Xiao F.P. (2017). Performance characteristics of terminal blend rubberized asphalt with sbs and polyphosphoric acid. Constr. Build. Mater..

[33] Ma T., Wang H., He L., Zhao Y.L., Huang X.M., Chen J. (2017). Property characterization of asphalt binders and mixtures modified by different crumb rubbers. J. Mater. Civil. Eng..

[34] Juganaru T., Bombos M., Vasilievici G., Bombos D. (2015). Devulcanized rubber for bitumen modification. Mater. Plast..

[35] Guo L., Lv D.J., Ren D.H., Qu L.N., Wang W.C., Hao K.F., Guo X.R., Chen T.C., Sun J.Y., Wang C.S. (2021). Effectiveness of original additives in waste rubbers for revulcanization after reclamation with a low-temperature mechanochemical devulcanization method. J. Clean. Prod..

[36] Valdes C., Hernandez C., Morales-Vera R., Andler R. (2021). Desulfurization of vulcanized rubber particles using biological and couple microwave-chemical methods. Front Environ. Sci.-Switz..

[37] Zhang B., Chen H.X., Zhang H.G., Kuang D.L., Wu J.Y., Zhang X.L. (2019). A study on physical and rheological properties of rubberized bitumen modified by different methods. Materials.

[38] Zhang B., Chen H.X., Zhang H.G., Wu Y.C., Kuang D.L., Guo F.J. (2020). Laboratory investigation of aging resistance for rubberized bitumen modified by using microwave activation crumb rubber and different modifiers. Materials.

[39] Wang W.S., Cheng Y.C., Tan G.J., Liu Z.Y., Shi C.L. (2018). Laboratory investigation on high- and low-temperature performances of asphalt mastics modified by waste oil shale ash. J. Mater. Cycles Waste.

[40] Zhou P.L., Wang W.S., Zhu L.L., Wang H.Y., Ai Y.M. (2021). Study on performance damage and mechanism analysis of asphalt under action of chloride salt erosion. Materials.

[41] Zheng W.H., Wang H.N., Chen Y., Ji J., You Z.P., Zhang Y.Q. (2021). A review on compatibility between crumb rubber and asphalt binder. Constr. Build. Mater..

[42] Geng J., Chen M., Xia C., Liao X., Chen Z., Chen H., Niu Y. (2022). Quantitative determination for effective rubber content in aged modified asphalt binder. J. Clean. Prod..

[43] Thives L.P., Pais J.C., Pereira P.A.A., Triches G., Amorim S.R. (2013). Assessment of the digestion time of asphalt rubber binder based on microscopy analysis. Constr. Build. Mater..

[44] Liu S.J., Zhou S.B., Peng A.H., Xuan W.A., Li W. (2019). Analysis of the performance and mechanism of desulfurized rubber and low-density polyethylene compound-modified asphalt. J. Appl. Polym. Sci..

[45] Zhao M.Z., Dong R.K., Chi Z.H., Aljarmouzi A., Li J.R. (2021). Effect of process variables on the chemical characteristics of crumb rubber desulfurized by waste cooking oil and its desulfurization mechanism. Constr. Build. Mater..

[46] Liu X.H., Bi Y.F., An Y.N., Zhou S.Q., Li F. (2021). Secondary-reaction method to improve performance of a compound modified rubber asphalt after short-term aging. J. Transp. Eng. Part B Pavements.

[47] Yi X.Y., Dong R.K., Tang N.P. (2020). Development of a novel binder rejuvenator composed by waste cooking oil and crumb tire rubber. Constr. Build. Mater..

[48] Lv S.T., Ma W.B., Zhao Z.G., Guo S.C. (2021). Improvement on the high-temperature stability and anti-aging performance of the rubberized asphalt binder with the lucobit additive. Constr. Build. Mater..

[49] Zhang J.W., Chen M.Z., Wu S.P., Zhou X.X., Zhao G.Y., Zhao Y.C., Cheng M. (2021). Evaluation of vocs inhibited effects and rheological properties of asphalt with high-content waste rubber powder. Constr. Build. Mater..

